# High-Quality Draft Genome Sequences of Crenobacter cavernae Strain CAVE-375 and Oxalobacteriaceae sp. Strain CAVE-383, Two Bacteria Isolated from Dripping Water in a Karstic Cave in Portugal

**DOI:** 10.1128/MRA.00127-19

**Published:** 2019-03-21

**Authors:** Catarina Coelho, António Veríssimo, Igor Tiago

**Affiliations:** a Centre for Functional Ecology, Department of Life Sciences, University of Coimbra, Coimbra, Portugal; Georgia Institute of Technology

## Abstract

Crenobacter cavernae CAVE-375 and Oxalobacteriaceae sp. strain CAVE-383, two Gram-negative bacteria, were isolated during the first microbiology survey performed in a karst cave in Portugal. We report here the release of their high-quality draft genome sequences, which will be useful for geographic-ecological purposes and the description of novel taxa.

## Abstract (precis)

Crenobacter cavernae CAVE-375 and Oxalobacteriaceae sp. strain CAVE-383, two Gram-negative bacteria, were isolated during the first microbiology survey performed in a karst cave in Portugal.

## ANNOUNCEMENT

A microbiological survey was performed in Algar do Pena cave, located in a karst massif known as Maciço Calcário Estremenho, in Central Eastern Portugal. Of several isolates, two strains (CAVE-375 and CAVE-383) were considered good candidates to represent novel taxa based on the amplified 16S rRNA gene sequence phylogenetic results. Their phenotypic characteristics were investigated and their whole genomes sequenced. Recently, the description of the novel species Crenobacter cavernae (1) within the family Neisseriaceae was released (GenBank accession number CP031337). Strain CAVE-375 showed the greatest phylogenetic affiliation to the recently described species, but despite the 16S rRNA gene similarity value of 99% between the two strains, the DNA-DNA hybridization (DDH) value determined for the sequenced genomes by the Genome-to-Genome Distance Calculator (2) was 62.66% (interval of 60.4 to 66.1%, determined by using formula 2). This value provides evidence that we are in the presence of a distinct species of Crenobacter cavernae, although the phenotypic characteristics are essentially the same (data not shown). The fact that these two strains, CAVE-375 and Crenobacter cavernae K1W11S-77^T^, were isolated from similar environments (karstic caves), but in two distant countries (Portugal and China, respectively) makes the release of the genome of strain CAVE-375 relevant for geographic-ecological studies.

Strain CAVE-383 showed the highest 16S rRNA gene phylogenetic affiliation to species of genus Herbaspirillum belonging to family Oxalobacteriaceae, with 16S rRNA gene similarity to the type strains of that genus ranging from 96.6 to 97.4%. While its correct phylogenetic affiliation is still being determined, the DNA-DNA hybridization values, calculated as above, were determined against the 150 genomes belonging to the family Oxalobacteriaceae available at NCBI GenBank, some of type strains and others from isolates. The DDH values determined ranged between 19 and 33% similarity (determined by using formula 2), providing evidence that strain CAVE-383 represents a new taxon within this family. The assembled genome, annotation results, and phenotypic results will be used to discriminate strain CAVE-383 from close relatives and provide data to describe it as the type strain of a novel genus and/or species within the family Oxalobacteriaceae.

Both strains were grown on nutrient agar (NA) at 25°C, and genomic DNA was extracted with an NZY microbial gDNA isolation kit (NZYTech, Portugal). Libraries were prepared using the Nextera XT library prep workflow (Illumina), and 2 × 150-nucleotide (nt) paired-end reads were generated on an Illumina MiSeq instrument. Quality trimming was executed using the sliding-window operation in Trim Galore! v0.5.0 (3) with default parameters. The final assembly was performed using the SPAdes v3.5.0 (4) assembler with default parameters and kmers of 33, 55, and 77 nt. The assembly was subjected to binning with MetaBat v2.12.1 (5) with default parameters, and a quality check was performed on the final resulting file using CheckM v1.0.12 (6) with default parameters.

For strain CAVE-375, 17,325,372 high-quality raw sequences were assembled into 15 contigs with an *N*_50_ value of 323,281 and a total genome size of 2,273,143 bp (2.9 Mb). Annotation using the NCBI Prokaryotic Genome Annotation Pipeline (PGAP) identified 2,779 protein coding sequences and 63 tRNA sequences. The whole genome has a G+C content of 65.9%, with a theoretical coverage value of 300×.

For strain CAVE-383, 10,289,999 high-quality raw sequences were assembled into 11 contigs, an *N*_50_ value of 1,195,780, and a total genome size of 4,119,171 bp (4.2 Mb). PGAP annotation identified 3,603 protein coding sequences, 4 rRNA sequences, and 46 tRNA sequences. The whole genome has a G+C content of 60.3%, with a theoretical coverage value of 300×.

### Data availability.

The whole-genome shotgun projects have been deposited at DDBJ/ENA/GenBank under the accession numbers REGP00000000 and REGR00000000 for strains CAVE-383 and CAVE-375, respectively. Strains are available from the authors upon request. Raw sequencing reads for strains CAVE-383 and CAVE-375 are available in the NCBI Sequence Read Archive under the accession numbers SRR8442863 and SRR8495389, respectively.
